# An Observational, Cross-Sectional Study to Investigate Whether Room Air Ventilators, Used in the Community Setting, Are Colonised by Potential Airborne Pathogens (IPAP Study)

**DOI:** 10.3390/jcm14041171

**Published:** 2025-02-11

**Authors:** Alison Armstrong, Ben Messer, Caroline Cullerton, Mark Lowes, Karen Heslop-Marshall, Allison Sykes, Stephen Wright, Anthony De Soyza

**Affiliations:** 1 North East Assisted Ventilation Service, The Newcastle upon Tyne Hospitals NHS Trust, Newcastle upon Tyne NE1 4LP, UKmark.lowes@nhs.net (M.L.);; 2 Population Health and Sciences Institute, The Medical School Newcastle University, Newcastle upon Tyne NE2 4HH, UK

**Keywords:** non-invasive ventilation, airborne pathogens, respiratory infection, decontamination

## Abstract

**Background/Objectives:** Long-term ventilation (LTV) is a widely used treatment for the management of patients with chronic respiratory failure. As use increases, it generates further questions about aspects of care. One issue is the potential risk of contamination within the device itself and the potential risk of respiratory tract infections in a subsequent user. Using an observational cross-sectional study design, the primary objective of this study was to identify whether airborne bacterial and fungal pathogens are present within a NIPPY 3+ (Breas Medical Ltd., Stratford Upon Avon, UK) room air ventilator following use in a community setting. **Methods:** Microbiological samples in the form of one single charcoal swab were taken from two specified areas of the device’s internal airflow pathway. **Results:** A total of 243 ventilators were sampled. A total of 215 ventilators with complete data collection were included in the study. A total of 84 (39%) were identified as having no growth and 131 (61%) were positive for bacterial and/or fungal growth. Overall, 307 organisms were grown from 131 ventilators ranging from 1 to 6 organisms per swab. Of the 215 ventilators screened, 15 (7%) grew organisms considered to be pathogenic. Well-established human pathogens were considered as ‘potentially pathogenic’ in this study due to the limitation of not obtaining patient-specific data, meaning host–pathogen interaction could not be determined. Of these, 14 grew one pathogenic organism and 1 grew four distinct pathogens. This is the largest study to date exploring the potential presence of airborne pathogens in room air ventilators. We have demonstrated that 61% of these devices were positive for bacterial or fungal growth and 7% were pathogenic. Pathogenic organisms included *Pseudomonas aeruginosa*, *Staphylococcus aureus* and *Aspergillus* sp. Although the growth of pathogenic organisms was relatively rare, there are important potential adverse clinical outcomes in patients with diseases commonly treated by LTV services. **Conclusions:** We have shown that the contamination of devices is rare, but, in 7%, there is contamination with potentially pathogenic organisms, which, if proven to be transferred between patients, could be a cause of worse patient outcomes.

## 1. Background

Long-term ventilation (LTV) is a widely recognised treatment for the management of patients with chronic respiratory failure. Since the 1980s, non-invasive ventilation (NIV) has been effectively delivered outside of the traditional hospital setting [1], using smaller and more portable positive pressure ventilators [2], with the treatment applied via a face mask. In their systematic review conducted in 2014, Hannan et al. provide good evidence that home ventilation improves both quality of life and mortality outcomes [3].

This treatment is now commonly used, and, as its use increases, it generates further questions about aspects of care. One issue is the potential risk of contamination within the device itself, potentially driving contamination back into the primary user; another issue is the potential risk of cross infections in any subsequent user of the ventilator.

The ventilators used in the community are considered ‘room air ventilators’, drawing air from the surrounding environment to deliver to the patient. These ventilators are not single-use devices and are reused between patients. The devices are predominantly those with a single-limb circuit design. In theory, this means that the airflow travels in one direction only, from device to patient. The expectation is that any exhaled air from the patient, which may potentially contain airborne pathogens, is exhausted into the environment via an expiratory port either within the interface (mask) or the ventilator circuit. The patient interface (mask and tubing) and the expiratory port are single-patient-use and therefore should pose no risk in terms of cross contamination. However, the potential for contamination within the ventilator itself from the environment in which it has been used has never been formally tested on a large scale.

Antibacterial (AB) filters are often applied in the acute clinical setting to provide a barrier to potential contamination between the device and the patient. However, they are not universally used in the home environment due to the potential to interfere with the pressure and alarm sensitivity of the device (Williams and Nunez 2019—conference abstract) [4]. A survey to explore national practice revealed that only 50% of LTV services in the United Kingdom use AB filters as a standard (Armstrong and Messer 2020—conference abstract) [5]. The British Thoracic Society’s guidance from 2002 recommends a bacterial filter for acute NIV but also states that “there is no airflow from the patient back into the ventilator” [6]. However, we have demonstrated in a recent bench study that exhaled gas does reach the ventilator and therefore may contaminate the device [7].

There are data regarding the incidence of respiratory tract infections in patients receiving long-term non-invasive ventilation. This field is complex given many of the indications for LTV are in themselves associated with increased respiratory infection rates, either as lower respiratory tract infections and/or pneumonia. Data from small series suggest that respiratory infection rates can be as high as 47–52% soon after treatment [8,9].

Previous studies have investigated the risk of device contamination. One study performed nasal swabs and also swabbed the ventilator mask and tubing of 40 LTV users [10]. Potentially pathogenic bacteria were isolated from six ventilator masks (15%) and the nasal swabs of 10 patients (25%). However, Mutagi and colleagues conducted a small study (seven devices) within a regional Cystic Fibrosis unit [11]. No evidence was found for pathogenic microbial contamination of the NIV devices, though most devices grew environmental organisms. A larger study found no increased risk of infection with the use of reprocessed CPAP devices, which are also room air turbine-driven devices, compared to newly issued devices [12]. This study also found evidence of contamination by environmental organisms. However, these studies used a variable study methodology; therefore, it remains unclear whether the incidence of chest infection in the studies described by Chenoweth [8] et al. and Campbell and Messer [9] is a direct result of contamination from a room air ventilator.

Currently, there is no standardised technique for the decontamination or disinfection of turbine-driven positive pressure ventilators that are reused between patients in the community setting.

This demonstrates a knowledge gap in the delivery of home LTV. The clinical teams responsible for the provision of care and those in the manufacturing industry have expressed concern about the lack of guidance around decontamination procedures. The current evidence is either out of date or out of context and fails to address the potential risk to this vulnerable patient group. Following an extensive review of the available literature, this is an area that requires further evidence to support clinical practice guidelines.

This presented an urgent need to explore whether the presence of respiratory tract pathogens exists within room air ventilators that might contribute to the respiratory infection morbidity seen in this vulnerable patient group.

## 2. Study Aim

Using an observational cross-sectional study design, the primary objective of this study was to identify whether airborne bacterial and fungal pathogens are present within a room air ventilator following use in the community setting. A secondary objective was to determine whether ventilators that had a positive bacterial growth had been in the patient’s home environment for longer than ventilators that had no bacterial growth, or if there was a correlation based on the number of previous users.

## 3. Methodology

### 3.1. Study Setting

The service provides care for adult patients across Northern England with a patient caseload at the time of the study of over 1000. Common indications for LTV are neuromuscular disease, chronic obstructive pulmonary disease and obesity-related respiratory failure.

### 3.2. Patient and Public Involvement

Patients were not involved in the study itself, but patient and public contributions informed the development of the initial study proposal. Opinions were sought on the research question and the requirement for consent, and advice was given on the patient information for subsequent dissemination of the findings.

### 3.3. Ethical Approval

Local Caldicott approvals for database use were sought and provided. The hospital review board gave approval for study conduct. National ethics approvals were deemed not to be required.

### 3.4. Routine Ventilator Care

Patients are provided with air inlet (dust particle) filters and asked to change these in accordance with manufacturers’ recommendations. Each ventilator is serviced routinely, usually every 12 months. If a patient has a ventilator change/service, the machine is serviced without any internal decontamination. Similarly, any returned ventilators are serviced without decontamination of the airflow pathway and reused for the next patient.

### 3.5. Ventilator Sampling

Expert advice from microbiology specialists was sought on the sampling procedure and also from the manufacturer with regard to the airflow pathway inside the device itself. The ventilators sampled were the most widely used ventilators within this service, which was the NIPPY 3+ (Breas Medical Ltd., Stratford Upon Avon, UK).

**Figure 1:**
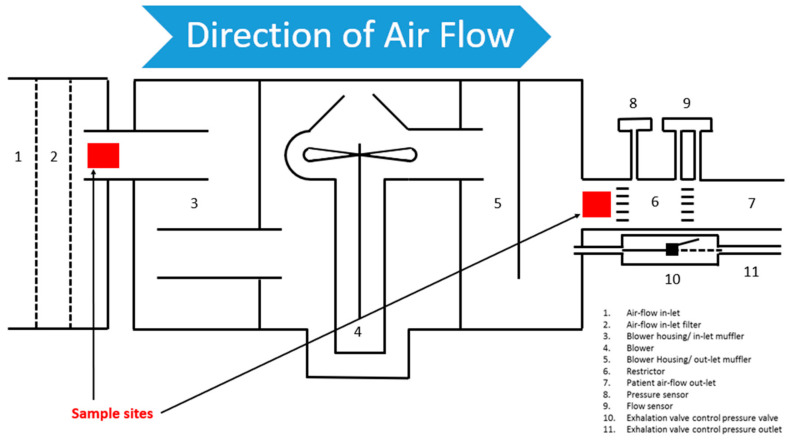
Sample sites.

Microbiological samples were taken from two specified areas of the device’s internal airflow pathway (Figure 1) using the same charcoal swab for both sites. It was felt that separate swabs to identify precise data about where potential contamination might occur would not add value, given that the airflow pathway is not protected between the inlet and the patient.

- Inside surface of the air inlet filter.
- Inside of the airflow outlet.

**Figure 2:**
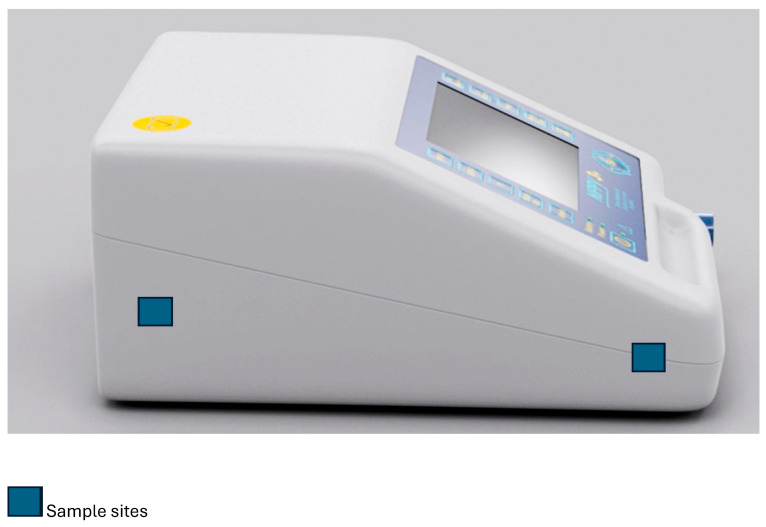
Sample site image.

The first sample site (leftward point) is internal to the ventilator downstream of a dust particle filter. The second sample site (rightward point) is also internal to the airflow pathway, prior to the airflow outlet. Figure 2 shows an image to contextualise the diagrammatic representation.

The sampling took place at a single point in time, when the device was being routinely reviewed by the Medical Electronics department. This would have been either during routine service, following equipment failure, or when the device was being returned to the department for any other reason. To ensure sampling processes were standardised, a limited number of personnel were identified and trained to conduct the swabbing process. We interrogated our electronic records to identify and record the number of days in the last environment (patient home) of each device and the total number of users of that device prior to the swabs being taken. This was to allow an understanding of whether the microbial growth increases over time, or if there was any link between microbial growth and the number of previous users of the device.

### 3.6. Microbiology Analysis

Ventilator swabs were mixed vigorously in 1 mL of sterile water after which 50 µL of this suspension was inoculated onto a range of culture media supporting the growth of respiratory, environmental and other organisms, as shown in Table 1.

Following incubation, culture plates were read daily with all distinct colony types being identified. Bacterial and Candida identification was carried out using the Bruker Matrix-Assisted Laser Desorption Ionisation—Time of Flight (MALDI-TOF). Conventional methods were used for the identification of filamentous fungi to a minimum of species (sp.) level. Colony-forming units were recorded and multiplied to provide CFU/mL for each organism present.

The study methodology was developed prior to the COVID-19 pandemic; therefore, screening for viral contamination was not part of the study protocol.

Testing all the NIPPY 3+ ventilators within clinical service, which stood at over 800 at the point of commencing the study, was not feasible. The intention was to give an estimated prevalence by sampling a proportion of all ventilators. To achieve a 95% confidence interval of ±5%, with an estimated five percent of ventilators colonised, a sample size of 214 ventilators was required [14].

Statistical analysis of categorical data was performed using either a chi-squared test or Fisher’s exact test, and analysis of normally distributed, continuous data was performed using Student’s t-test. Statistical significance was defined as *p* ≤ 0.05.

All statistical tests were performed using Microsoft Excel 365.

## 4. Results

**Figure 3:**
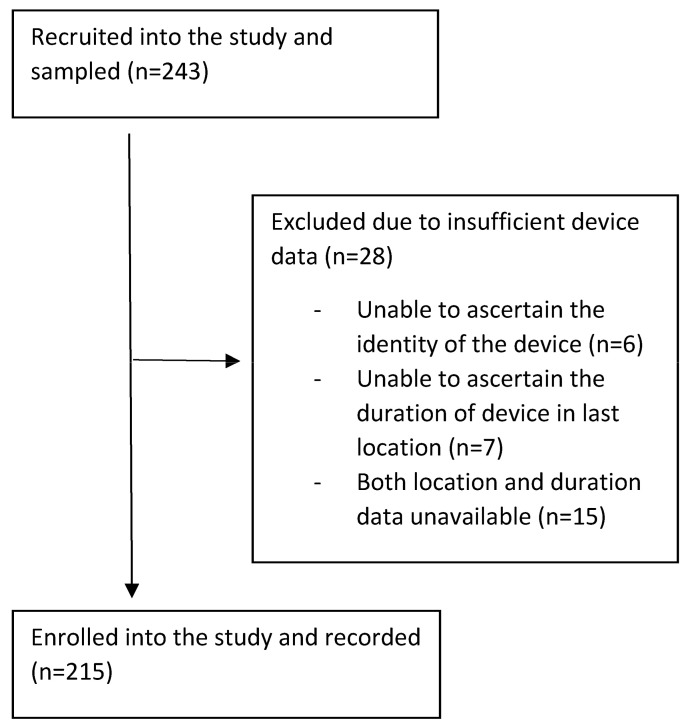
Number of ventilators sampled.

It was not possible to derive all of the information required for some of the devices sampled due to omissions in the historical records; therefore, a slightly larger number of devices were sampled than originally planned to allow for some missing data. A total of 243 ventilators were sampled, with 215 included in the study with complete data collection (Figure 3).

The median number of previous users of each device was 2 (range 1 to 18). The mean amount of time each device spent in the last environment prior to swabbing was 218 days (range 9 to 4059 days).

**Figure 4:**
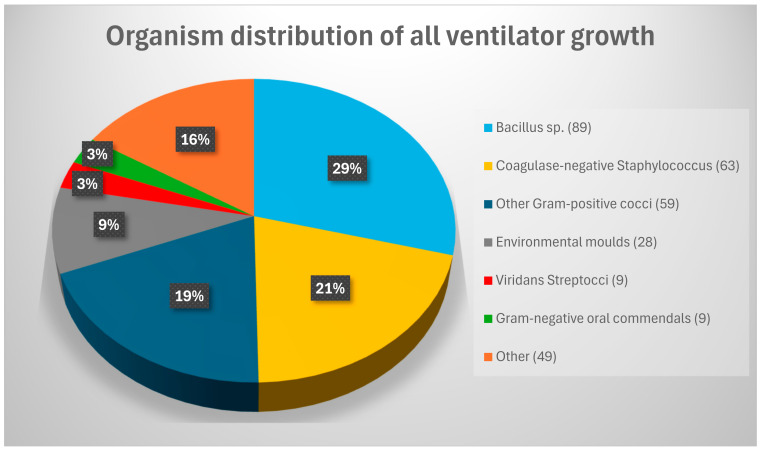
Frequency of organisms isolated. Other Gram-positive cocci included *Micrococcus* sp. *Dermacoccus* sp. and *Kocuria* sp. Environmental moulds included *Penicillium* sp., *Paecilomyces* sp., *Chaetomium* sp. and non-sporing moulds. Gram-negative oral commensals consisted of non-pathogenic *Neisseria* sp. and *Moraxella* sp. ‘Other’ consisted of *Aspergillus* sp. (8), Enterobacterales (8), Yeasts (7), *Corynebacterium* sp. (7), environmental *Pseudomonas* sp. (5) including *P. stutzeri*, *P. oryzihabitans*, *P. putida group and P. monteili,i* Mucoraceous mould (3), *Acinetobacter* sp. (1), *Mycobacteria fortuitum* (1), *Pseudomonas aeruginosa* (1), *Staphylococcus aureus* (1), *Stenotrophomonas maltophilia* (1), *Sporosarcina* sp. (1) *Roseomonas* sp. (1), *Brachybacterium* sp. (1) *Ewingella* sp. (1), *Novosphingobium* sp. (1) and *Paracoccus* sp. (1). The species breakdown is presented in Table 2.

Of the 215 ventilator samples cultured, 131 (61%) were positive for bacterial and/or fungal growth and 84 (39%) had no growth. Overall, 307 organisms were grown from 131 ventilators ranging from 1 to 6 organisms per ventilator swab. CFU/mL ranged from 20 to >2000 with an average CFU/mL per positive ventilator of 253. The organisms grown are presented in Figure 4.

Most frequently isolated organisms included *Bacillus* sp., Coagulase-negative Staphylococcus, other Gram-positive cocci such as *Micrococcus* sp. and environmental fungi such as *Penicillium* sp.

Of the 215 ventilators screened, 15 (7%) grew organisms considered to be pathogenic; of these, 14 grew a single pathogenic organism and 1 grew four distinct pathogens. Of the pathogenic organisms, the CFU/mL of each isolate ranged from 20 to >2000, with an average CFU/mL of all organisms of 236 per ventilator (illustrated in Table 2).

There was no association between the number of previous users of a device and the presence of detectable bacteria grown from the swab. The median number of previous users of the devices that had no growth was 2 and the median number of previous users of devices that grew airborne pathogens was 1. However, there was a relationship between the length of time the device was in the last environment prior to being swabbed and the presence of airborne pathogens. Those with no airborne pathogens were significantly more likely (*p* = 0.0055) to have been in the same environment for longer (mean 1102 days) than those with organisms present (mean 697.5 days).

As a result of guidance during the COVID-19 pandemic, devices recruited after March 2020 were placed into a 5-day quarantine period prior to transfer to Medical Electronics. This would have delayed the time between leaving the patient environment to the swab being taken by at least 5 days. Prior to the quarantine period, 76 ventilators were screened; of these, 20 (26%) were found to have no growth and 56 (74%) were positive for bacterial/fungal growth with an average CFU/mL of 185. Following the introduction of the 5-day quarantine period prior to the swab being taken, 139 ventilators were screened. Of these, 64 (46%) were found to have no growth and 75 (54%) were positive for bacterial/fungal growth with an average CFU/mL of 138. The differences between positivity rates prior to and following the introduction of the device quarantine period were statistically significant (*p* = 0.0054).

## 5. Discussion

This is the largest study to date exploring the potential presence of pathogens in room air ventilators. We have investigated the contamination of the NIPPY 3+ room air ventilator following use in a community setting, with complete data collection on 215 devices applying a novel methodology sampling both the inlet and outlet chambers. This captured potential environmental contamination on the inlet and also patient–ventilator contamination on the outlet.

We have demonstrated that 61% of NIV devices were positive for bacterial or fungal growth and 7% were potentially pathogenic. Pathogenic organisms included *Pseudomonas aeruginosa*, *Staphylococcus aureus* and *Aspergillus* sp. Although the growth of pathogenic organisms was relatively rare, there are potentially important adverse clinical outcomes in patients with diseases commonly treated by home ventilation services. In bronchiectasis and COPD, the presence of *Pseudomonas aeruginosa* is associated with exacerbation frequency [15], hospital admissions [16], worse quality of life [17] and increased risk of mortality [18]. The presence of *Staphylococcus aureus* in patients with Cystic Fibrosis (CF) is considered one of the main causes of recurrent chest infections and progressive decline in lung function [19]. Invasive pulmonary aspergillosis in patients with COPD is associated with a poor prognosis [20]. These are all recognised indications for LTV. In most cases, we found low numbers of organisms, although a select few had higher bacterial loads.

There was a broad range of organisms isolated in our study. The most common respiratory pathogens isolated from the general population were *Streptococcus pneumoniae* and *Haemophilus influenza* [21]. Neither of these was isolated from this study, but it is recognised that *Haemophilus influenzae* is more fastidious and that our microbiological sampling technique and later quarantine approach may have had some impact on the culture of these pathogens.

There was a somewhat surprising negative relationship between the duration of the placement of the ventilator in a single patient environment and bacterial colonisation. The reasons for this observation are not clear. It is possible that certain conditions such as obesity-related respiratory failure and kyphoscoliosis are associated with greater stability and less potential for contamination. Patients with conditions such as COPD, CF and Bronchiectasis are generally treated with LTV due to advanced disease; therefore, they are at a greater risk of instability and mortality. As such, they may die sooner after the commencement of NIV than patients with more stable disease and therefore the ventilators, which are more likely to be contaminated, may be in circulation for a shorter period of time, which may account for this negative correlation.

This was the first study that swabbed the airflow pathway through the ventilator from the air inlet to the ventilator outlet. Other studies have swabbed sites such as the mask and tubing, which are single-use and therefore should not pose a risk of cross contamination.

These results are consistent with those of Rodriguez, who found that 15% of NIV circuits were contaminated [10]. This is higher than our 7% growth rate of pathogenic organisms, but Rodriguez swabbed the mask and ventilator tubing, which are closer to the patient and more likely to become contaminated. Steinhauer investigated 50 CPAP machines that had been used by patients [12]. This study also noted contamination with environmental organisms and that potentially pathogenic organisms were less common. They did not report the percentage of devices that were contaminated. These results are not consistent with the study of Mutagi, who investigated seven ventilators from patients with CF and found no pathogenic bacteria [11]. However, most devices grew environmental organisms and the small sample size may have reduced the ability to detect pathogenic organisms. Furthermore, sampling sites were different in our study, which sampled the ventilator internally, whereas Mutagi sampled externally. In addition, ventilators in our study were from patients with diverse diagnoses. Finally, two devices in the Mutagi study were sterilised with ethylene oxide prior to swabbing, and swabbing of the device was not performed for at least seven weeks.

There are some limitations in our study that should be acknowledged. Our methodology combined the swab sites, so we cannot report the differential rates of contamination nor the associations between inlet and outlet contamination. As our study was focused on the device, we did not report the clinical indication for the ventilator. The environmental contamination and cross infection risks linked with CF are well documented and likely different to those of a patient requiring ventilation for obesity-related respiratory failure.

We therefore cannot refute that some of the potentially pathogenic organisms isolated are a consequence of patient-to-environment contamination with subsequent ventilator contamination. This may minimise the impact of ventilator contamination on the patient, as the patient’s own airway bacterial load will likely be significantly higher. However, there remains a concern that the ventilator, when applied to a different patient, may lead to a new airway infection. Pseudomonas cross infection is well described in CF; several outbreaks have been described, and some are linked to fomites/environmental acquisition rather than direct patient–patient contact. This is important as we have previously reported a rate of Pseudomonas in our LTV population of nearly 18% [22].

Other limitations are that we did not systematically record nor sample any ventilator-associated humidification systems. Respiratory humidifiers are often used alongside non-invasive ventilation. It is well recognised that water sources (taps, etc.) can become contaminated with Pseudomonas, leading to outbreaks [23]. Also, a control group was not sampled, and it is possible that some environmental contamination may be present when the device is newly supplied by the manufacturer. However, the organisms identified were those that often affect patients with conditions that would benefit from home mechanical ventilation, making it more likely that these results are from patient contamination.

For some devices, it was impossible to be certain about the number of previous users. This occurred when a device was loaned to the physiotherapy team for acute use and potentially applied to one or several patients. For the purposes of the study, this episode was counted as one patient. Therefore, the number recorded was a minimum number of previous users. However, as described, the number of previous users appeared to have no impact on the presence of organisms. In addition, there were a small number of occasions where the device location was not recorded. This left gaps in the chronological location record. To ensure full data were available on all devices studied, we continued to sample devices until the target number of 214 was reached with the required data available, but we acknowledge that a more sophisticated multivariate analysis would have strengthened our conclusions.

In the only guidance available, Tablan et al. recognise that their recommendations are applicable to devices used in acute care and hospital settings and are therefore not transferrable to home NIV settings [24]. Holdcroft et al. state that the internal machinery of mechanical ventilators is not considered an important source of bacterial colonisation [25]. They conclude that sterilisation or high-level disinfection of the internal components is unnecessary. A local Decontamination of Reusable Medical Devices Policy recognises that in order to maintain Care Quality Commission (CQC) registration, trust in maintaining appropriate levels of cleanliness and hygiene in relation to reusable medical devices is required; however, it gives no advice with regard to the decontamination of turbine-driven positive pressure ventilators. More specifically, the Decontamination of Healthcare Equipment following Patient Use and Prior to Service or Repair local policy refers to national legislation (Health and Safety at Work Act 1974) [26] and (Control of Substances Hazardous to Health Regulations 2002) [27] but is focused more on protecting staff rather than patients. It only gives generic guidance on the process of surface decontamination.

Currently, there is no recognised process for internal device decontamination, and these results present evidence supporting further exploration of this issue. Further work is required to understand whether the presence of airborne pathogens in the device leads to cross contamination and subsequent infections and whether the introduction of antibacterial filters or a set quarantine period as a standard practice would reduce the risk to a subsequent user. Finally, the study protocol was developed prior to the COVID-19 pandemic. Many domiciliary devices were recommissioned during the pandemic for acute use. It is important to investigate viral contamination of ventilators/devices that would inform infection control practices and isolation requirements for devices during acute usage for pneumonitis due to COVID-19 or other respiratory viruses.

We demonstrated a statistically significant decrease in positivity rates following the quarantine period introduced due to the pandemic. This may be relevant in terms of recommendations for practice if taking a device out of service for a short period of time reduces the risk of potential device contamination. Additionally, if there is cross contamination of viruses/bacteria, further work is required to understand the optimal time for quarantine.

## 6. Conclusions

We have shown that there is evidence of device contamination of ventilators used in a community setting. Whilst the presence of potentially pathogenic organisms is rare (7%), this could result in worse clinical outcomes for patients using LTV services if they are proven to be transferred between patients. Further work is needed to understand whether there is a connection between the presence of airborne pathogens in the device and subsequent infections. Longitudinal studies with repeated sampling of both ventilator- and patient-derived samples should be considered in future studies.

## Figures and Tables

**Table 1:** Culture media and growth requirements.

Culture Medium	Supplier	Incubation		
		Temp °C	Atmosphere	Time
Columbia blood agar	In-house preparation	37	Aerobic	48 h
Columbia blood agar	In-house preparation	30	Aerobic	48 h
Chocolate blood agar	In-house preparation	37	CO_2_	48 h
CPSE	bioMérieux	37	Aerobic	48 h
Sabouraud dextrose agar	In-house preparation	37	Aerobic	5 days
Rapid-growing Mycobacteria agar	In-house preparation	30	Aerobic	28 days

Columbia blood agar and chocolate blood agar were made using Columbia agar powder (Oxoid, Basingstoke, UK) supplemented with 5% defibrinated horse blood (TSC Biosciences, Buckingham, UK). Sabouraud dextrose agar was made using Sabouraud dextrose powder (Oxoid, Basingstoke, UK). Rapid-growing Mycobacteria agar was made as previously described [13].

**Table 2:** Pathogenic organism growth (20 CFU/mL unless otherwise stated).

Device Reference	Pathogenic Growth			
	Organism 1	Organism 2	Organism 3	Organism 4
1	*Aspergillus flavus*	-	-	-
4	*Aspergillus nidulans*	-	-	-
12	*Acinetobacter* sp.	-	-	-
20	*Pseudomonas aeruginosa* >2000 CFU/mL	*Klebsiella pneumoniae* 160 CFU/mL	*Stenotrophomonas maltophilia* 700 CFU/mL	*Mycobacterium fortuitum* 120 CFU/mL
43	*Aspergillus fumigatus* 140 CFU/mL	-	-	-
49	*Aspergillus* sp. 40 CFU/mL	-	-	-
66	*Aspergillus candidus*	-	-	-
69	*Candida* sp.	-	-	-
76	*Rhizopus* sp.	-	-	-
86	*Staphylococcus aureus* 160 CFU/mL	-	-	-
120	*Aspergillus fumigatus*	-	-	-
123	*Aspergillus fumigatus*	-	-	-
149	Mucoraceous mould	-	-	-
159	*Rhizopus* sp.	-	-	-
216	*Aspergillus niger*	-	-	-

## Data Availability

The raw data supporting the conclusions of this article will be made available by the authors on request.
